# Cutaneous Melanoma in the Context of Aging

**DOI:** 10.3390/medicina61122115

**Published:** 2025-11-27

**Authors:** Monica Neagu, Carolina Constantin, Sabina Zurac

**Affiliations:** 1 ”Victor Babes” National Institute, 99-101 Splaiul Independentei, 050096 Bucharest, Romania; caroconstantin@gmail.com; 2 Colentina Clinical Hospital, 19-21 Soseaua Stefan cel Mare, 020125 Bucharest, Romania; sabina_zurac@yahoo.com; 3 Faculty of Biology, University of Bucharest, 91-95 Splaiul Independentei, 050095 Bucharest, Romania; 4 Department of Pathology, “Carol Davila” University of Medicine and Pharmacy, 8 Bld. 12 Eroii Sanitari, 050474 Bucharest, Romania

**Keywords:** ageing, skin cancers, melanoma, immunotherapy

## Abstract

Ageing is sustained by a complex network of cellular and molecular mechanisms. The main mechanisms are cellular senescence, telomere attrition, gene expression changes, metabolic dysregulations, oxidative stress and epigenetic modifications such as DNA methylation. All these networks can harbor the initiation of age-related diseases, skin cancer included. The studies published in the last years linking ageing and skin cancers focus on basal and squamous carcinomas, melanomas and Merkel cell carcinomas. Our review will focus on skin melanomas as one of the aggressive skin cancers along with Merkel cell carcinomas. Several long-term studies conducted on large populations have shown that in elderly individuals melanoma related to photo-exposure has doubled in the last decade. The clinic-pathological pattern of skin melanomas is different in aged patients and is guided also by immune-related mechanisms. Besides sun exposure, metabolic deregulations and obesity can be risk factors in melanomas. Controversial results were published on obesity risk in melanomas; however, the adipose tissue favors increased cytokines and growth factors production contributing to melanoma aggressiveness. Moreover, immunotherapy that is not offered in geriatric patients as often as in young ones has proven to be as efficient as in younger ones, although the aged-related co-morbidities can impede the immunotherapy choice. Without being exhaustive, our review has synthesized current research and critically assessed the links between aging as a normal physiological process to the initiation and propagation of skin cancers, focusing on cutaneous melanoma. The review highlights the differences at various levels of skin melanoma developed in aged patients compared to younger one and gives the general outlines for diagnosis, prognosis and therapeutical approaches in aged patients.

## 1. Introduction

Ageing is a complex process driven by intrinsic factors like heredity and extrinsic factors like lifestyle and environment. There are age-related decline of various systems and their functions over time that can favor the initiation of pathologies like cancer, diabetes, cardiovascular diseases, neurodegenerative disorders and so on. A complex network of cellular and molecular mechanisms is driving ageing, such as cellular senescence, telomere attrition, gene expression changes, metabolic dysregulations and epigenetic modifications. Within epigenetic alterations DNA methylation (DNAm) has an important role in ageing. All these complex networks can harbor biomarkers of ageing and the age-related diseases [1]. Degenerative normal processes include skin alterations, musculoskeletal disorders, cardiovascular diseases, diabetes, neurodegenerative disorders, and cancer. Furthermore, there are common points between cancer and ageing, as both arise from genetic and epigenetic alterations accumulation [2].

The increased life expectancy, particularly of those aged over 70, due to significant medical advances made over the last century especially in high-income countries, will lead to an increased number of pathologies related to ageing. It is estimated that the global population of people over 80 years will be approximately 430 million by 2050, three times larger than the population registered in 2019 [3]. Skin cancers are the most commonly diagnosed cancer in humans; moreover, the increased skin cancer incidence in aged population is increasing. This is probably due to a longer duration of exposure to external factors such as ultraviolet radiation (UV) and pollution and by intrinsic factors, like decreased repair mechanisms, increased metabolic stress and oxidative stress. These intrinsic and extrinsic factors increase DNA damage and mutation that can lead to carcinogenesis. Interestingly normal skin has the highest mutational load compared to all other tissues and these mutation burden exponentially increases with age, skin site, sun-damage history, and skin photo type [4]. Moreover, physiological ageing decreases the efficiency of immune surveillance and immune responses against cancer. These hindered immune mechanisms sustain a chronic level of systemic inflammation favoring various pathologies [5].

In the last ten years over 2300 papers with the key words “skin cancer” and “ageing” were retrieved from PubMed, MEDLINE and Scopus databases. These papers focus mainly on basal and squamous cell carcinomas, skin melanomas (SM) and Merkel cell carcinomas. Without being exhaustive, our review will present the latest up-dates in the processes that link ageing with skin cancers, focusing on skin melanomas as one of the most aggressive skin cancers. We have synthesized current research and critically assessed the links between ageing as a normal physiological process and the initiation and propagation of skin cancers, focusing on SM. The review highlights the differences identified at various in aged patients diagnosed with SM in comparison to younger patients and give the outlines for diagnosis, prognosis and therapeutical approaches in aged patients.

## 2. Populational Ageing and Skin Cancers

There is accelerated incidence of skin cancers as one in every three diagnosed cancers is a skin cancer, thus currently, 2–3 million non-melanoma skin cancers and over 130,000 melanoma skin cancers are diagnosed globally each year out of 10 million cases of diagnosed cancers. As data are appending to national registries and there are still extended numbers of countries without up-dated registries it is most possible that these figures are under estimated. Additional increases of skin cancers incidence are expected in Europe for the coming decades. In Caucasian populations, since the 40s both SM and keratinocyte cancers (KC) have been rising dramatically and depending on the registry, the rise is from 15 to almost 50-fold. In terms of unfortunate leadership, Europe is ranking first in the number of UV-related cancer patients. As reviewed by Brochez et al. in 2025 citing Globocan 2020 and 2022 data, Europe has the highest incidence in melanomas and the second in non-melanoma KCs [6].

Major skin cancers in terms of incidence but also as high mortality percentages are the non-melanoma cancers (NMSC), SM and Merkel cell carcinomas. Although NMSC are the most common KC as it accounts for 7 out of 8 new cancers diagnosed in Australia, SM and Merkel cell carcinomas are not as common but they sustain the main percentages of mortality due skin cancers in Australia. As mentioned, skin cancer risk factors include intrinsic and environmental factors, out of which UV and advanced age are the major factors. Within NMSC, basal cell carcinoma (BCC) is the most common malignancy in Caucasians, its incidence is increasing by 2% per year. Squamous cell carcinoma (SCC) is the second most common skin cancer, with an incidence of approximately 100 per 100,000 persons. Main risk factors for BCC and SCC are light skin color, UV radiation exposure, and chronic immunosuppression. SM incidence in Australia is around 50 cases per 100,000 persons [7]. As in the case of SM, NMSCs incidence is also increasing. In a study evaluating SCC in Japanese population, it was shown that in people over 80 years the NMSC increased from 14.7 to 51.6/100,000 people between 2007 and 2016 [8].

In a 2024 published report the pathological profile of aged SM patients was evaluated. In over 1300 SM patients with ages over 80 years and adults under 65 years of age, patient data was recorded in 2017 by the Regional Veneto Cancer Registry (Northeast Italy). In aged patients SM was associated with the presence of ulceration, a higher Breslow index, a lower prevalence of tumor-infiltrating lymphocytes, and a more advanced pathological tumor-node-metastasis staging (pTNM) stage at presentation. Thus, the clinic-pathological presentation of SM in over 80 years patients has a distinctive profile and hence can significantly influence the (immuno)-therapy applied [9]. A similar study has shown that another parameter in aged population is the histological type of SM. Analyzing over 80,000 cases it was shown that the relative survival after diagnosis is strongly mediated by tumor thickness [10]. Jourdain et al. have shown in aged population that while the average Breslow index is 4.9 mm in patients over 80 and statistically higher that in younger patients, the BRAF (B-Raf proto-oncogene, serine/threonine kinase) mutation status is a characteristic of young SM patients and not of older ones. The study collected and investigated the data of 344 patients in 2018–2022 [11]. When critically evaluating these findings, we can highlight that different histology parameters that were observed in aged melanoma patients can be due to a delayed presentation for diagnosis.

**Figure 1:**
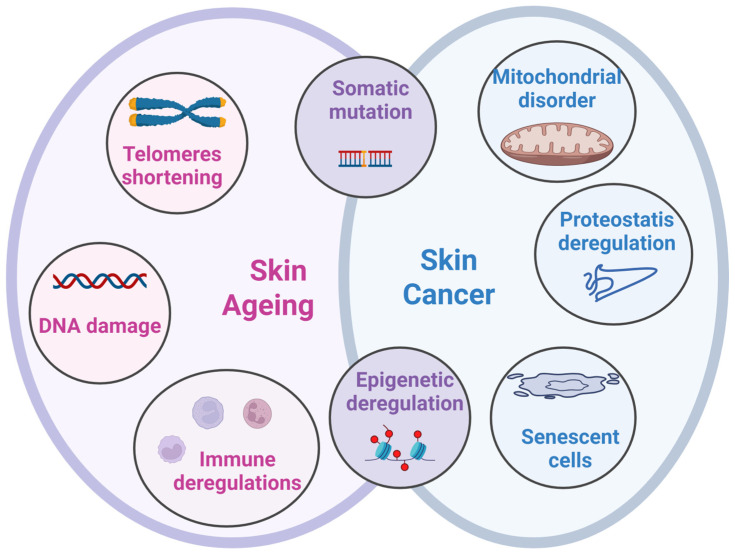
Specific and common pathways for ageing and cancer. Skin ageing is characterized by telomere shortening, DNA damage, immune deregulations, while skin cancer is characterized by senescent cell, proteostasis deregulations, and mitochondrial disorders. Both phenomena, skin ageing and skin tumorigenesis, are induced by somatic mutations and epigenetic deregulations, processes that overlap in the physiological status of ageing or in the pathological status.

Actinic keratosis (AK), xerosis cutis, and inflammatory diseases are frequently diagnosed dermatoses in elderly. These skin conditions are pre-requisite states for NMSC and SM cancers [12]. These skin conditions can precede NMSC and SM cancers [12]. The overall inflammatory status of the skin is a condition that precludes skin carcinogenesis. In aged patients this inflammatory status is prolonged favoring skin tumorigenesis. A study published for German population that have extrapolated a 2030 forecast has shown that SM incidence will steadily increase in aged individuals with the steepest increases for patients ≥60 years, especially for males [13]. Skin ageing is a physiological process but sun exposure can accelerate it, increasing thus skin cancer risk. Genetic traits also govern this skin ageing process, therefore, in a study investigating 22 genome-wide significant loci for women and men, important correlations with skin ageing were found. Further investigation into the genetics of skin aging by sex (male, female) would contribute to understanding the genetic factors of both skin aging and skin cancer. An interesting finding was shown, namely the association of gene deregulations found in aged skin with gene associated to male-pattern baldness [14]. Several studies have shown that skin health in the elderly is seriously affected, constituting a priority of geriatric healthcare system. Thus, in Australian populations it was reported that among the 200 investigated adults almost 60% had severe or moderate-to-severe photo-damage on the majority of their body sites. All individuals with severe photo-damage were over 50 years, had a high incidence of a past skin cancer, and were smokers [15]. In Germany a cohort of over 100 hospitalized geriatric patients were examined for dermatological diseases, namely precancerous skin lesions and epithelial skin cancer. After examination there were found precancerous skin lesions in 20% in males compared to almost 7% in females and epithelial skin cancer in 34% vs. 13.3%, respectively [16]. These studies pin-point once more that added factors increase the genetically-based ageing process of the skin. Looking at the reported percentages of skin damaged in elderly patients it can be concluded that the geographical site is as well important in term of UV exposures. For example, Australia reports a 60% damaged skin in elderly, in comparison to Germany that reports around 30%. Therefore, there are processes that intersect between skin ageing and tumorigenesis as schematically presented in Figure 1.

## 3. Skin Melanoma in Aged Population

Only in the last two years, 2024–2025, around 50 papers were published having as subject melanoma and ageing. Skin diseases in the aged population are frequent but the clinicopathologic correlation has to distinguish among various aged-induced pathologies with or without relation to the skin cancer [17]. Analyzing over 30,000 patients with confirmed SM, several risk factors were investigated, such as skin photo-damage, coffee intake, smoking habits, alcohol consumption and socioeconomic status. SM risk was associated with the ease of skin tanning and with childhood sunburn history. Therefore, it has been shown in children that cumulative sun exposure for more than 2 weeks per year leads to sunburn, and if the UV flux during exposure increases by 10% on an annual average, this would lead to a 19% increase in the chances of developing melanoma in adulthood. The other factors were not significantly associated with SM risk such as coffee intake, alcohol consumption, smoking and socioeconomic status [18]. A similar study performed on around 2000 patients that were supervised for 30 years has shown that the location of SM is preponderantly on the photo-exposed skin. There is an increased incidence of head and neck melanomas in both sexes while a decrease in trunk melanomas localization in men, and a reduction of the lower extremities’ localization in women. All the results of this long-time study revealed that the phenomenon of ageing doubled by skin’s photo-exposure definitely contributes to the pathogenesis of SM [19]. As in other types of skin cancers, in SM, environmental factors drive the skin tumorigenesis and enhance with the ageing of the individuals. In Northeast Italy it was reported, after evaluating almost 1400 SM patients, the relation of natural ageing and the incidence of this skin cancer. Three age groups were evaluated––over 80 years, 65–79 years, and adults under 65 years of age. The clinic-pathological pattern of SM is different in aged patients, for example a lower prevalence of tumor-infiltrating lymphocytes, characteristics that should be taken into account when refining (immuno)-therapeutic procedures [9]. Moreover, in a retrospective study it was shown in almost 1000 patients that immunotherapy with anti-PD-1 (Programmed cell death protein 1) is particularly recommended for melanomas originating from chronically sun-exposed areas [20]. These later studies show that the response of the immune system to a developing SM is not identical in aged and young patients and that the therapeutical approaches should be refined in association with the age of the patient and with the associated co-morbidities.

### 3.1. Genetic and Epigenetic Traits in Aged Melanoma Patients

There are several important studies that delineate the genetic/epigenetic background of SM, but far less when depicting the genetic make-up of aged SM patients. Various models were studied from in silico, in vitro to actual SM samples. An in-silico model studied the melanoma-associated genes focusing on germline mutations, somatic mutations, and genome-wide association that identified melanoma risk genes. The study showed that melanoma risk network is associated with mechanisms appending to DNA metabolism, telomeres, DNA damage and repair, cellular ageing, and response to UV irradiation. Moreover, this in silico model could predict the anti-tumoral effect of phytochemicals, harmine and berberine chloride, in in vitro cellular models using SK-MEL-28 human melanoma cell line [21]. Over 50% of skin cancers appear after 65 years and cellular senescence is a process associated with ageing, but its relation with a specific genetic pattern is still a subject of study. Patients with Familial Melanoma Syndrome (FMS) have germline defects in the CDKN2A gene. This gene is encoding a cellular cycle protein regulator, inhibitor of cyclin-dependent kinase 4 (p16INK4a). When melanocytes from FMS patients were investigated it was found that these cells express significantly less p16INK4a but express higher levels of the DNA-damage protein *γ*H2AX (H2A histone family member X). Though, skin fibroblasts have also genetic defects having an increased replicative capacity and abnormal nuclear morphology. Patient derived fibroblasts also secreted less senescence-associated secretory phenotype (SASP) than control cells. Individuals with FMs have a high incidence of developing SM and this is probably due to the dysregulation of senescence in at least two major cells, melanocytes and fibroblasts. Moreover, these deregulations hinder T cells recruitment reducing skin cutaneous immunosurveillance [22]. Fibroblasts from aged patients have extracellular vesicles (EV) carrying different cargo. Thus, ageing EV have reduced tetraspanin CD9 expression. CD9 knockdown induced a significant increase in angiopoietin-349 like protein 2 (ANGPTL2), an angiogenesis promoter. These data indicate that aged EVs are promoting a tumor-permissive microenvironment [23] and that they are mirroring the genetic and epigenetic accumulation in aged tissues [24]. In UK it was reported that around 10% of SM patients have a family history of this tumor, therefore the familial genetic melanoma is a clear risk factor. The study revealed that FMS is linked mainly to CDKN2A, RB1 (RB Transcriptional Corepressor 1), and telomerase reverse transcriptase (TERT) mutations, genetic deregulations that are associated with the attenuation of cell senescence [25].

UV radiation damages DNA, hence the nucleic acid is changing its gene expression. In an in vitro experimental model, it was shown that around 50% of the investigated genes have significant changes one day post-UV irradiation. These deregulated genes sustain photo-ageing damage and further skin cancer initiation; moreover, the findings prove that UV-induced damages are profound at genetic level [26].

In contrast with SM that has a sun-exposure trigger, uveal melanomas and blue nevi, are driven by mutations in the intracellular signaling pathway of cysteinyl leukotriene receptor 2. These mutations drive the growth of melanocytes in one tissue [27], but can inhibit the growth of melanocytes in blue nevi [28]. The assertion is extremely meaningful as tissue environment can control the balance between uncontrolled cell growth and senescence [26].

Another molecular mechanism that is activated by chronic UV irradiation and is further inducing mutations, was identified in the Transforming Growth Factor (TGFβ) signalling pathway. Its activation induces the initiation of collagen degradation through metalloproteinase (MMP)-activation and photo-inflammation. If UV irradiation is chronic, it can induce mutations in genes encoding for TGFβRI, TGFβRII, Suppressor of Mothers Against Decapentaplegic (SMAD)2, and SMAD4. These TGFβ signalling pathways mutations empower tumour cells to escape from TGFβ-induced growth inhibition, induce genomic instability and activation of cancer stem cells; all these conjoint processes leading to skin cancers. The accumulated mutations cause TGFβ overexpression in the tumor microenvironment (TME) of various skin cancers, including NMSC and SM. TGFβ overexpression propagates inflammation, angiogenesis, cancer-associated fibroblasts (CAF), immune suppression, tumour evasion, and eventually metastasis. Treatments that target TGFβ over-expression can be important therapeutical options for both photo-ageing and skin cancers [29].

In terms of epigenetic signatures, SM can have alterations in DNAm, RNA methylation, histone modifications, noncoding RNAs (ncRNAs), leading to altered proteins expressions and functions [30]. Thus, it was reported that deregulated gene promoters that are a characteristic for ageing are also characteristic for cancer, namely there is a global DNA hypomethylation in both processes. Analyzing over 2000 samples from The Cancer Genome Atlas (TCGA) data base it was shown that almost 100,000 cytosine––guanine base (CpG) sites were found differentially methylated in ageing while almost 300,000 in cancer. Hereafter the methylation changes (hyper- or hypomethylation) have similar genomic distribution in ageing and cancer. Moreover, hypermethylated regions in both ageing and cancer have a similar chromatin signature, while hypomethylated DNA sequences were identified in different chromatin regions. Thus, hypomethylated sequences in ageing are mainly in the activating histone posttranslational modification H3K4me1 (Histone 3), while in cancer, were associated with the repressive H3K9me3 [31]. In 2025 a database was compiled by Baskaran et al. showing the proteomic and genomic deregulations induced by UV. Almost 1000 genes, 500 proteins, over 50 metabolites and, from the epigenetic domain, almost 80 miRNAs were found similarly involved in cancer and ageing [32].

We can draw an outline for SM in ageing highlighting that if the genes that have increased rate of mutations accumulate in both ageing and tumorigeneisis, the epigenetic mechanisms govern mostly the already established TME in SM driving tumor expansion and metastasis.

### 3.2. Cellular Senescence and Immunosenescence in Melanoma

Ageing per se is a phenomenon that inevitably affects all living organisms and that transcends to all the tissues and organs. Owing to the accumulation of DNA damage and telomere erosion, aged cells will be irreversibly arrested in the G1 phase of their cell cycle, a process known as replicative senescence [33]. Skin ageing is a process associated naturally with the loss of fibrous tissue and a slower cell turnover; these processes are associated also with decreased immune reactions to aggressors. Exposure to the environmental factors, mainly UV radiation accelerates the intrinsic ageing and photo-ageing interferes with skin biology, increasing the risk of skin carcinogenesis.

Understanding the underlying molecular mechanisms is gaining increased importance in the last years. Senescent cells within the aged skin are characterized by irreversibly arrested growth, reduced or absent proliferation, specific gene expression and hyper-secretory secretory pattern, and are actively involved in several age-related diseases, skin cancer included [33,34].

Cellular senescence in cancer prevents the uncontrolled cell proliferation favoring their clearance. Nevertheless, in cancer there is a subtle balance in the senescence mechanisms. It was shown in the elderly patients that skin aggression and poor clearance of injured cells increase cancer incidence. When tumor cells develop, they can activate skin fibroblasts into a pro-tumorigenic phenotype that facilitates cancer progression [35,36]. Using single-cell RNA sequencing and spatial transcriptomics Yu et al. have published in 2025 the evaluation of senescent cells within the ageing skin. Photo-ageing was associated with an increase of senescent cells, matching the chronological ageing of the skin. The team has identified a skin-specific cellular senescence gene set, associated to photo-ageing, chronological ageing, and non-replicating Cyclin-dependent kinase inhibitor (CDKN) 1A+ (p21) cells. Senescent melanocytes have elevated melanin synthesis in epidermis, while in the dermis, senescent reticular dermal fibroblasts have decreased collagen and elastic fibre synthesis. In photo-aged skin the spatial analysis has revealed the tendency for senescent cells to cluster, a process sustaining the senescence splicing program and genome reorganization [37]. Smith and Carroll have recently shown that the molecular mechanisms of cellular senescence have as central player the dysregulation of the master growth regulator, Target of Rapamycin Complex 1 (mTORC1) interacting with lysosomes and that this deregulation governs the senescence phenotypes [38].

In SM an interesting domain is the study of senescence in benign melanocyte tumors, specifically, the melanocytic nevi. In a mouse model it was shown that nevi have an arrested status developed due to cell interactions [39]. In a study published by us in 2024 we have shown that although melanocytic nevi have a high mutational load, cell cycle proteins have a high expression in comparison to melanoma, this process sustaining the arrested cell cycle [40]. Nevi are comprised of senescent cells with dysfunctional telomeres, having also a minor non-senescent cell subpopulation [39,41].

The immune system is ageing along with the entire organism. Highlighted by Doherty et al. in 2025 immunosenescence is driven by alterations in both the number and the relative balance of T- and B-lymphocytes populations [42]. These changes show the reduction of naïve adaptive immune cells, the increase of memory T-cells, and the reduced ability to respond to new aggressors. No reduced T cell proliferation or capacity of the antigen presenting cells (APC) is associated. Furthermore, age-related changes appear also in the innate immune system, reduced chemotaxis, reduced cytotoxicity, and abnormal cytokine production [42]. Ageing induces the decli8ne of Tregulatory cells, resulting in a weakened immune system. Therapeutical approaches that preserve Tregulatory cell’s function could decelerate the immunosenescence process [43]. We have also reported in around 300 healthy individuals that there are age-related immune changes in both adaptive and innate immune cells, and for both humoral and cellular immune parameters. While decreased total T lymphocytes, T-CD8+ and NK cells were found associated with healthy ageing, increased T-CD4+ and B cells were identified. An interesting finding was reported for the neutrophils cellular populations where healthy ageing induced an increased inflammatory pattern [44]. Neutrophils populations still are an underexplored domain in both physiological ageing and pathology. Investigating the ageing immune system in a melanoma animal model, using RNA sequencing it was shown that physiological ageing induces in the lung a microenvironment dominated by an increased proportion of IL-17-expressing γδT and neutrophils. This immune inflammatory pattern actually is preparing a pre-metastatic niche. The expansion of γδ17 is due to the age-dependent downregulation of the receptor S1pr1, that is an immune trafficking receptor. Additionally, expanded γδ17 recruit neutrophils with specific markers expression (e.g., low CD62L, high CXCR4–C-X-C Chemokine Receptor Type 4), these characteristics being predominant for tumor-promoting neutrophils. The aged-dependent inflammatory milieu, neutrophils included, suppresses the anti-tumoral roles of CD8+ T cells. When modulating this aged-induced alterations of the immune system interesting results were obtained. Depletion of γδT or of neutrophils reduces metastasis in aged animals. Moreover, administering procyanidin C1, a senolytic agent, the aged-dependent tumor-promoting effects were reduced [45]. Wicaksono et al. have shown in in vitro experimental models using melanoma cell lines that natural anti-tumoral and anti-inflammatory compounds can target both receptors associated with melanoma (e.g., GRP78—78-kilodalton glucose-regulated protein, IRE1–Inositol-requiring enzyme 1, BRAF) and ageing pathways/receptors (e.g., mTOR, AMPK–AMP-activated protein kinase, SIRT1–Sirtuin 1). Compounds extracted from *Caulerpa racemosa* were tested on melanoma A375 cell line exhibiting BRAF-V600E mutation and on B16-F10 murine melanoma cell lines. Out of all compounds, caulersin, modulated mTOR, AMPK, and SIRT1 pathways and downregulated GRP78, IRE1 and BRAF signaling [46].

In 2024 the immunosenescence index (ISI) was published with the aim to identify novel biomarkers and new targets in SM [47]. Integrated bioinformatics identified risk prognostic genes, and multi-omics analysis pin-pointed the 314 possible biomarkers. SM patients that displayed a lower ISI associated a better survival rate along with several genetic traits like low chromosomal instability, low somatic copy number variations (CNVs), low somatic mutations, higher immune infiltration, and increased sensitivity to immunotherapy. Single-cell analysis has shown that higher ISI was explicitly expressed in monocytes, correlating with the differentiation of monocytes in SM [47].

Immunosenescence, as reviewed by Rodriguez et al., is a process of continuous transformation of the elements of the immune system that leads to a lessened ability to set up an efficient innate and adaptive immune response. Immunosenescence is characterized by an increased highly differentiated T lymphocytes populations while naive T are decreased. Hence immunosenescence can account for various aged-related pathologies, as the incidence and prevalence of most cancers increase with age [48].

### 3.3. Ageing at the Sub-Cellular Level

Noticeable, all the sub-cellular level organelles will suffer from the ageing process. The central player of the molecular deregulation resides mainly in the mitochondrion system. Mitochondrial deregulations would induce an unequilibrated balance oxidation–antioxidation, mitochondrial DNA (mtDNA) damage and mitophagy. There are several processes initiated by deregulated mitochondria. Excessive reactive oxygen species (ROS) generation, would induce cell damage and death. Increased inflammation will take place via the activation of damage-associated molecular patterns (DAMPs), inflammasomes and activation of inflammatory cells. Ageing associates with dysfunction in energy metabolism, genetic instability, with oxidative stress and inflammation [49]. Therefore, mitochondria deregulation is associated with tumorigenesis. In an experimental animal model, using B16F10 murine melanoma, cells that are used for standard experimental induction of SM [50], the metastatic process was studied in the aged animals’ context. Similarly to other tumors, mitochondrial biology in melanoma cells is deregulated, and the cells exhibit increased glycolysis and decreased oxidative phosphorylation, an adaptation for hypoxic conditions that are found mostly in metastasis [51]. A recent study showed an interesting approach of using mitochondria to induce tumor cells destruction. When normal mitochondria were inoculated in melanoma metastasis bearing animals decreased tumor growth rate and increased survival in melanoma-animals was obtained. Moreover, when normal mitochondria isolated from young animals were inoculated, the effect was enhanced in comparison to aged-mitochondria. The normal mitochondria interfered with tumor cell metabolisms, reduced glycolysis and increased oxidative environment, while increasing tumor cell apoptosis, necrosis, and mitophagy [52]. Indeed, natural ageing features also the functional decline of our intra-cellular organelles [53].

Another cellular organelle that was studied in the context of senescence is the proteasome compartment. The proteasome is an important cellular machinery and the age-related disruption of proteostasis induces the accumulation of abnormal and less functional proteins. Thus, Piskorz et al. have shown in 2024 that proteasome inhibitors can induce senescence in melanoma cell lines. A proteasome inhibitor-marizomib was assessed in A375 and G361 melanoma cell lines. Cellular senescence in G361 was present after 4 days of treatment, namely increased SA-β-galactosidase, IL-8, P-P53 (tumor antigen p53) expressions, increased G2/M and S phases of cell cycle, decreased lamin B1. In A375 cell line the treatment induced increased apoptosis, subG1 phase, P-P53, cleaved lamin B1. Thus, while some melanoma cell lines have reduced cellular senescence, other react with reduced apoptosis, showing once more the clear heterogeneity of melanoma tumor cells [54]. SASP of melanoma cells is a feature of cellular senescence. Various therapeutical approaches can modulate SASP, such as metabolic interventions and on mitochondrial energy metabolism. The DNA methylating agent, temozolomide, can increase the level of fusion proteins mitofusin (Mfn) 1 and 2 in melanoma, moreover it can, through silencing Mfn1 or Mfn2, reduce the expression and secretion of IL-6 by senescent cells. Using shotgun proteomics, the impact of silencing Mfn1 on the SASP was investigated. By silencing Mfn1 several immunomodulatory molecules were deregulated, including galectin-9. The work of Tarallo et al. has delineated that methylating agents would add to the melanoma therapeutical approaches. Moreover, mitochondrial dynamic molecules can be future pharmacological targets modulating the SASP in melanoma [55].

### 3.4. Other Factors Enhancing Ageing Processes

Several various processes and mechanisms were found associated to SM development and ageing from various areas: neuroendocrine system, metabolic disorders, tumor microbiome, TME characteristics and so on. Even circadian rhythm influences at genetic levels the initiation of SM as it is known that circadian clock is heavily implicated in the etiology of metabolic diseases, chronic inflammation and cancers. Thus, it was recently published that circadian rhythm genes (CRGs) relate to SM. Using weighted gene co-expression network analysis and overlapping CRGs, the study identified 125 melanoma-related CRGs genes. Out of these 125 CRG genes, six were identified as important in melanoma diagnosis and prognosis, e.g., ABCC2 (ATP-binding cassette subfamily C member 2), CA14 (Carbonic Anhydrase 14), EGR3 (Early Growth Response 3), FBXW7, (F-box and WD repeat domain containing 7), LDHB (Lactate Dehydrogenase B), and PSEN2 (presenilin 2)**.** These CGR could be important predictors of melanoma pathogenesis [56].

Marino-Bravante et al. have studied in aged SM patients the extracellular matrix (ECM) protein hyaluronan and proteoglycan binding link (HAPLN)1. HAPLN1 maintains the integrity of melanoma-associated neoangiogenesis, proving increased collagen and VE-cadherin expressions. The vascular permeability is increased in this case by HAPLN1 that induces ICAM1 to internalize VE-cadherin. Authors show that if ICAM1 is blocked, the tumor size is reduced in animal model of aged mice. The role of ICAM1 in aged SM patients can be explored as therapy targets hindering tumorigenesis [57].

Extra-cutaneous factors have an important influence on SM development. Recently metabolic deregulations and obesity were denominated as risk factors. The adipose tissue harbors various functional alterations contributing to many diseases, including cancer. The increase of adipose stromal/stem cells (ASCs) impacts melanoma progression. ASCs in association with increased cytokines and growth factors production, leads to SM metastasis. Thus, besides the sun exposure as risk factor, lifestyles can induce skin cancer. Melanocortin pathway appending to both melanin synthesis and obesity is linking these apparently two unrelated conditions. Adipokines, secreted by the adipose tissue, are directly linked to inflammation, being involved in angiogenesis, migration, invasion and proliferation of melanoma cells [58]. We have shown in SM patients that the chronic inflammatory state is modulated by the metabolic imbalance, and that among the adipokines, leptin (LEP) is increasing with melanoma staging and the age of the patients. Using protein microarray technology, we have shown in SM patients, that out of all the 42 investigated cytokines and chemokines, the LEP circulatory level has the highest value [59]. Controversial results were published on obesity risk in SM. The “obesity paradox”, a notion developed in the last years, focusing on the immune check point inhibitors (ICI) therapy efficacy in SM patients has shown that an increased body mass index (BMI) is linked to improved clinical results after ICI [60]. One explanation of the “obesity paradox” in SM patients could be given by Giampetri et al., namely the fact that SM patients have lower expression of 4 genes: CD36 (fatty acid translocase), MARCO (pro-tumor macrophage marker), FABP (Fatty acid-binding protein)4, and FABP7, compared to other cancer patients. Having a lower expression of CD36 and FABP4 genes, SM patients with increased BMI have actually a reduction of the cellular internalization of fat. Therefore, obese SM patients are less sensitive to a high dietary fat intake [61].

During ageing, another phenomenon can influence the SM development. Endocrine alterations induce an increase in fat mass, but also to the loss of bone, muscle mass and strength. Environmental and inflammatory statuses are added risk factors to the ageing-induced endocrine systems alterations [62]. Various studies suggest a role played by neuroendocrine factors acting directly on tumor cells, modulating their proliferation and metastasis capability, or indirectly through immune/inflammatory processes that impact SM progression. Our studies have shown that stress hormones directly influence melanoma cell proliferation [63].

Another risk factor in SM is the tumor microbiome involved in major process like oncogenesis, and (immune-based) treatment outcome. In a recent study, Dravillas et al. has used RNA sequencing (RNA-seq) to identify tumor microbiota. The immune-responders had an enrichment of bacteriophages from *Uroviricota phylum*, while non-responders had enrichment of mainly *Campylobacter jejuni*. These microorganisms were correlated with immune-related gene expression. The study points out an interesting association. The tumor microbiome and immune-related genes are robustly interacting and driving the efficacy of immunotherapy [64].

Another important process in cellular/molecular ageing is autophagy. It is a degradation mechanism involved in overall metabolism and cellular quality control, main processes in ageing. Within the physiological ageing, autophagy is decreased and is inversely associated with an increasing age. Cassidy et al. has shown in an animal model that if the process of autophagy is restored, a significant extension of lifespan is obtained. The experiment shows however another interesting outcome, namely in the autophagy-restored mice the spontaneous tumor incidence is increased. Probably this is the first mention of autophagy as having a biphasic role in oncology, thus being sequentially tumor suppressive and oncogenic [65]. Autophagy regulates cellular metabolic status by suppressing anabolism. Cellular metabolism deregulation is a hallmark of cancer, skin cancers included [66,67] but it is also associated to ageing [68]. Studies are still on-going to get insights into the autophagy triggered in energy-stressed cancer cells and how this phenomenon can be exploited in therapy, knowing that various metabolic pathways are involved. Moreover, as in ageing both mechanisms are deregulated, further understanding of these mechanisms could be beneficial toward the development of better cancer therapeutics.

**Figure 2:**
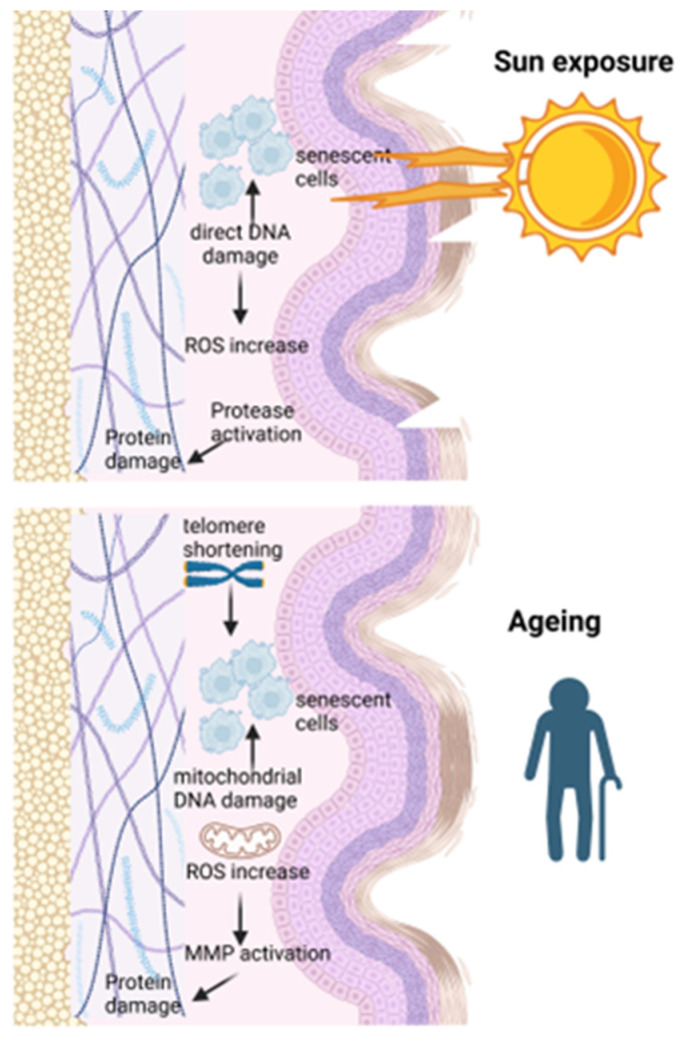
Main molecular traits of physiological skin ageing and of UV-induced skin ageing. Both processes have common or specific pathways. In upper panel, in sun-exposed skin, photo-ageing occurs by the direct action of UV irradiation, that is altering and damaging the DNA that induce cellular senescence. The DNA damage increases reactive oxygen species (ROS) generation and proteases activation that leads to protein destruction. In lower panel, in natural ageing, were all the tissues are ageing, telomeres are shortening, the cells become senescent when mitochondrial DNA is damaged. The mitochondrial functions are overall altered and the increase in ROS damages through oxidative stress induces once more the cellular DNA alteration and MMP activation that degrades extracellular matrix components. Both pathways set up the scene for tumorigenesis induction in skin, one through photo-ageing and the other through the overall ageing process.

As presented in Figure 1, another pivotal mechanism driving tumorigenesis and the immune-oncogenic escape is the shortening of telomeres accompanied by the activation of telomerase. Telomeres that are constituted by the long nucleotide repeats and the protein complex at chromosome ends, are essential molecular drivers of genomic integrity. Telomere length varies due to germline genetics and environmental exposures [69]. The process of telomere shortening that is accompanying normal ageing fosters genomic instability, chromosomal aberrations, increasing hence the risk of carcinogenesis [70]. Telomers’ length in SM patients harbor genetic instability traits that can increase the risk of developing SM [71]. The pathophysiology of telomere length in cancer is complex. It has the role of tumour suppressor via induction of apoptosis and cellular senescence, and a facilitator of carcinogenesis via genomic instability [72]. Recently Liu et al. has shown the anti-ageing strategies focusing on telomere protection through drug therapy and lifestyle adjustments [73]. Figure 2 outlines the main molecular mechanisms that govern skin’s (photo) ageing.

An integrative Table 1 presents the main characteristics of old vs. young SM patients.

## 4. Ageing Modulators Influencing Skin Cancer Initiation

As mentioned in the previous section, recent studies investigate the therapeutical potential of various compounds in the quest to limit/reduce skin ageing. As epidermis and dermis senescent cells were associated with dermatological diseases, emerging therapeutic approaches such as senolytics and senomorphics can provide solutions for alleviating skin ageing and senescence-associated skin diseases [82].

Cellular senescence modulation can be influenced by the use of natural bioactive compounds like resveratrol. This natural polyphenol can interfere and modulate various pathways like cell cycle, cell growth, apoptosis, senescence and inflammation. In vitro evaluation has shown that resveratrol’s effect depends on the cell types and biological contexts [83] and its complex action on cellular ageing and tumorigenesis was extensively reviewed [84]. The implications of resveratrol effect are more complex as gut microbiota promotes the synthesis of resveratrol from its precursor, thus regulating the microbiota composition aiding cancer prevention and slowing down ageing processes [85]. Recently it was shown that various other natural compounds (e.g., curcumin, epigallocatechin gallate, thymoquinone) among resveratrol target main pathways involved in cellular senescence and tissue degeneration, regulating key molecular players such as sirtuins, 5′ AMP-activated protein kinase (AMPK), Nuclear factor kappa-light-chain-enhancer of activated B cells (NF-κB), and mTOR [86]. For AK prevention various compounds were approved as around 10% of lesions can develop into invasive SCC. Among them, ingenol mebutuate gel extracted from *Euphorbia peplus* L., was shown to be very effective for treating AK with good tolerability, no systemic absorption, and excellent patient compliance [87]. But, after Health Canada’s review found evidence of skin cancer with the use of ingenol mebutate, the European Medicines Agency (EMA) reviewed its safety issue and in April 2020, concluded that ingenol mebutate may increase the risk of skin cancer. Hence on 11 February 2020, the manufacturer withdrew the product from the European Union market [88]. A natural extract from *Polypodium leucotomos* (Fernblock^®^, FB) has proven its antioxidant photoprotective properties with potent anti-ageing effect. In in vitro model of skin fibroblasts, it was shown that this extract prevents UV-induced cell damage, it reduces MMP-1 and cathepsin K expression and reduces the alterations of fibrillin 1, fibrillin 2 and elastin expression [89].

Endonuclease V (T4N5) and photolyases, have the abilities to remove cyclobutane pyrimidine dimers induced by UV irradiation. These molecules from the class of DNA repair enzymes can reduce precancerous skin lesions and further reduce the initiation of skin cancers. Topical application of T4N5 post UV exposure abrogated the upregulation of interleukin-10 and tumor necrosis factor alpha (TNFα), cytokines that induce immune suppression [90]. Topical DNA repair enzymes extracted from *Micrococcus luteus* and photo-lyase were applied in skin patches daily for 2 weeks post UV irradiation. The compounds did not have a significant effect on UV-induced gene deregulation as UV exposure causes acute changes, skin cancer growth and gene expression deregulation [91].

Preserving the normal physiology of the skin, dampening the natural ageing effects can be important steps to reduce the initiation of skin cancers.

## 5. Melanoma Therapy in Aged Patients

As described above, the immune system endures age-related alterations that influence both tumor initiation and progression. Immunotherapy in SM has changed the therapeutical paradigm in this deadly disease. Immune checkpoint molecules are having great success in prolonging the SM patient’s life, but the ageing process is influencing the immunotherapeutic approaches [92].

### 5.1. Immunotherapy in Aged Patients

Aged patients have added comorbidities and frailty, which may impact their ability to tolerate immune-related adverse effect and benefit from ICI or targeted therapy. In almost 900 SM patients with over 65 years recruited in 2018–2022 in Netherlands were subjected to ICI therapy. The study showed similar benefit of immunotherapy in aged patients. Nevertheless, aged patients with multiple comorbidities were at increased risk of multiple immune-related adverse effects and had a lower recurrence-free survival (RFS). This should be considered when deciding upon adjuvant treatment [78]. In contrast, the study of Jourdain et al. has shown no difference between younger and older patients in terms of adverse effect to ICI therapy [11]. The Dutch Melanoma Treatment Registry has shown on over 3000 SM patients stratified by over 65 years of age that patients aged over 75 years are less frequently treated. When they are treated there is no statistically significant increase in adverse effects and just a mild statistical PFS when compared to younger patients [93]. These differences can have various explanations, for example genetic differences and different mutational profiles of the tumor can be some of these explanations. Thus, the mutational profile of melanomas varies with age, so older patients tend to have more NRAS (NRAS proto-oncogene GTP-ase/Neuroblastoma RAS Viral (v-ras) Oncogene Homolog) mutations, while younger have more BRAF mutations [80].

In the protocol for establishing SM staging, sentinel lymph node analyses are needed according to the guidelines in order to access the therapies. Investigating the prognostic significance of sentinel lymph node positivity in aged melanoma patients it was shown that the presence of melanoma cells within the sentinel lymph node is not correlated with the overall survival but with the disease-specific survival [94]. In a study published in 2024 performed on French population it was shown that in aged population the therapeutical approach can be different. Sentinel lymph node evaluation was more frequently performed in patients under 80, while wide excision was performed more frequently in patients over 80. First-line treatment was more frequently performed in patients under 80, but no difference for the second and third lines of therapies [11]. Overall, it was shown that older SM patients have a lower incidence of sentinel lymph nodes metastasis compared to younger patients, but they have a higher mortality rate.

There are clear differences in SM patients depending on their biological sex. We have shown in experimental SM models that the immunological pattern and the clinical evolution is different in male compared to female animals [95]. In a more recent study, it was shown that melanoma incidence increases with age and in the male biological sex. Inherent immune response is dependent on the biological sex constellation and within the study it was depicted that skin fibroblasts suffer an age-mediated, sex-dependent alterations in terms of their proliferative capacity, senescence, ROS production and stress response. Therefore, fibroblasts isolated from male patients sustain a more invasive, therapy-resistant melanomas through an increased tyrosine kinase receptors AXL expression. Ageing in fibroblasts induced Enhancer of zeste homolog 2 (EZH2) decline and increased Bone Morphogenetic Protein 2 (BMP2) secretion, these mechanisms induce slower-cycling, highly invasive and therapy-resistant melanomas. Therapeutical inhibition of BMP2 activity reduces invasive phenotypes and sensitizes melanomas to BRAF/MEK inhibition [96].

Immunotherapy in SM aged patients has in general the same outlines like in younger patients with the specification that probably, compared to younger SM patients, the stage is more advanced when patients address the dermatologist/physician. However adverse effect can be more serious in aged patients, probably due to additional comorbidities that associate to ageing.

### 5.2. Predictors of Immunotherapy Efficacy in Older Patients

Similar to any other cancer types, in SM there is a continuous quest of good predictors for diagnosis, prognosis and therapy monitoring. Li et al. have published in 2025 a study evaluating predictive indicators in immunotherapy approaches. In various clinical trials encompassing over 13,800 SM patients the predictors for efficient immunotherapy were evaluated. In combination therapies, patients with age under 65 years had an improved OS and RFS, while aged patients over 65 years had only an improved OS. The predictors for efficient therapy were low baseline lactate dehydrogenase (LDH), Eastern Cooperative Oncology Group (ECOG) Performance Status = 0, and M stage at study entry. These parameters could depict the improvements in RFS and OS when receiving combination therapy in patients under 65 years of age. The study shows that male patients can have a larger clinical benefit from combined immunotherapy, assertion associated probably with tumor mutational burden (TMB) and estrogen levels. Male patients diagnosed with SM that have an increased age that are subjected to combination therapy have a good OS, but not a good RFS [97].

RNAs can be good predictors for both prognostic and therapy efficacy. Small nucleolar RNAs (snoRNAs) were assessed in melanoma samples and a prognostic model comprising snoRNAs was published in 2024. This pattern consists of 12 snoRNAs (e.g., SNORD9, SNORA31, SNORD14E, SNORA14A, SNORA5A, SNORD83A, SNORA75, AL096855, AC007684, SNORD14A, SNORA65, AC004839) correlated with the tumor immune infiltration. Moreover, analyzing the snoRNA pattern it was shown that it can indicate the efficacy for immunotherapy [98].

As presented above, epigenetic factors are involved in both tumorigenesis and ageing processes. In a recent study it was tested a telomerase activator as an epigenetic regulator in melanoma cell line model. Telomerase activator induced cellular senescence, histone deacetylases (HDAC8 and HDAC10) expression was upregulated, and Telomerase reverse transcriptase (hTERT) gene expression was down-regulated. Therefore, epigenetic factors can be therapeutically targeted in melanoma cells [99]. Within the epigenetic domain SETD2 (SET Domain-Containing Protein 2) is a histone methyltransferase that remodulates chromatin and regulates the genetic domains. Recently this epigenetic molecule emerged as a tumor suppressor. Xiong et al. have investigated using RNA-449 seq, melanomas clinical data and their immunotherapy response. SETD2 was found down-regulated in melanoma samples corroborated with an unfavorable survival rate [100]. SETD2 is a methyltransferase involved in several epigenetic mechanisms such as histone modification, DNA repair, mRNA regulation, genomic stability, alternative splicing, and IFN-α signaling [101]. Cells lacking SETD2 are more aggressive and invasive, resistant to chemotherapy, but more sensitive to tyrosine kinase inhibitors (TKIs). The study focusing on SETD2 dysfunction in melanoma proves that SM patients having this feature are directed toward immunotherapy as first therapeutical line [100].

Long noncoding RNA (lncRNA) were recently associated with tumor infiltrating lymphocytes (TILs) in SM. Macrophages that infiltrate the tumor are associated with specific lncRNAs (e.g., PART1, LINC00968, LINC00954, LINC00944, LINC00518, C20orf197) having predictive power for patient’s disease outcome. These lncRNAs correlated with various immune processes that take place in TME. Moreover, their pattern can predict immune checkpoint inhibitor therapy whether anti-PD-1 or anti-CTLA-4 (Cytotoxic T-lymphocyte associated protein 4) [102]. Hypoxia-related lncRNAs (HRLs) was also tackled by Liao et al. using MSigDB (Molecular Signatures Database) and TCGA databases. The authors established a risk model based on HRLs taking into account immune infiltration, TMB analysis, drug sensitivity and immunotherapy evaluation. These HRLs can be prognostic markers for the efficacy of the immune response and for the sensitivity to specific immunotherapy. Moreover, new therapeutic approaches targeting hypoxic TME, e.g., HIF-1α inhibitors, hypoxia relief and oxygen sensitive therapy can be developed in the future for SM patients [103]. Studying RNA to identify age-related transcriptome variations interesting results were shown. Genes like FOS, NR4A, and ITGB1 genes were significantly over-expressed in older SM patients that had also positive sentinel lymph node. IRAK3- and Wnt10b- were the main pathways associated with positive sentinel lymph node [104].

From the metabolome domain a recent study has shown that six serum metabolites can predict the diagnosis of SM in aged patients and that muramic acid is specifically correlated across melanoma stages [105].

Biomarkers that can be predictors for diagnosis, prognosis and therapy monitoring have a huge range from the proteomics, metabolomic, genomic and epigenomic areas, but there are still studies that need validation. We are convinced that there will be no “golden” biomarker in SM and that only a selected panel of molecules would bring in the future years a robust predictors panel.

## 6. Integrative Perspectives

Former knowledge at the beginning of the century, gave the sun exposure a therapeutic/prophylactic power in rickets, but knowledge evolved and now the UV component of sunlight has become a known carcinogen. Although skin formulations blocking UVB and UVA have minimized premature sun-ageing and skin cancer risk there is still a constant raise in skin cancers world-wide. Although mtDNA was discovered in the 1960s, the influence of UV irradiation upon DNA was recently found influencing both ageing and tumorigenesis [106]. The effect done by UV radiation upon DNA is the induction of photo- dimers at di-pyrimidine sites; these dimers hinder DNA replication and transcription inducing a general stress upon cellular physiology. Moreover, oxidative DNA lesions are induced by solar irradiation along with total breaking of the double strand in single strands. All these molecular deregulations impose on genomic integrity and are associated with ageing and diseases such as skin cancers. Generated mechanisms impede DNA damage repair, cell cycle, apoptosis, transcription and chromatin remodeling [107]. Age is the main risk factor for skin cancers, this type of cancer being the most frequent in aged individuals. The overall population has an increased age, thus the effective number of people developing skin cancers is increasing and an increased age is associated with a more advanced melanoma at diagnosis. Understanding the molecular networks that are involved in both natural ageing and cancer initiations is critical to improve patient outcomes. Ageing brings an accumulation of genetic and epigenetic alterations in organs that if extrinsic factors concur tumor cells are induced [108]. Concomitantly the immune system degenerates, thus tumor cell recognition by the immune system is flawed, contributing to advanced tumorigenesis [109]. Circulating immune cells have characteristics associated to ageing but were not associated with response to immunotherapy, a topic still open to further advanced studies [110]. While these findings suggest that the level of frailty and ageing may not necessarily preclude the efficacy of ICI therapy, further investigation is needed to fully understand the impact of frailty and ageing on immunotherapy. Population in general has an increased percentage of aged individuals and correlated with the increased UV radiation skin cancers these represent a major health challenge [111].

In recent years immunotherapy-based therapeutical approaches have improved the prognosis of melanoma patients. Nevertheless, as melanoma patients do not respond to immunotherapy- based strategies totally, around 50% responsiveness, therapies in aged individuals have to take into account new mechanisms that could be targeted.

Among various diseases, cancer is an ageing-associated disease, but the underlying molecular associations between ageing and tumorigenesis are still largely unknown. Skin ageing is a multifactorial process triggered by both intrinsic and extrinsic factors. Ageing is driven by a pleiades of processes such as oxidative stress, chronic inflammation, genomic instability, immune response deregulations, mitochondrial dysfunction and all these processes can initiate various skin cancers.

## Figures and Tables

**Table 1:** Main differences in aged SM patients compared to younger patients.

Parameter	Patients > 65 Years	Patients < 65 Years	References
Histology			
Breslow index	Mean 4.9 mm	Mean 1.9 mm	[9,11,74]
Ulceration	increased	decreased	[9]
Mitotic rate	High	Medium	[9,11,74]
TIL	Low	High	[9,75]
Preponderant tumor localization	Head and neck	Trunk and extremities	[74]
Subtypes frequency	Nodular melanoma and lentigo maligna	Superficial spreading melanoma	[76]
Nodal metastasis frequency	Low	High	[77]
Melanoma-specific survival	Low	High	[10,78,79]
Mutational pattern			
BRAF mutation frequency	Low	High	[11,80]
NRAS mutation frequency	High	Low	[11,80]
Immune pattern			
Naïve CD8 + T cells	Low levels	Normal levels	[5,22]
Tissue-resident effector memory CD8 + T cells	High levels	Normal levels	[42,43]
T cell proliferation and APC ability	NS	NS	[5,22]
Treg	Low levels	Normal	[42,43]
Chemotaxis, cytotoxicity, cytokine production	Reduced	Normal	[42,43]
Treatment response rates	NS	NS	[5,22]
Other cellular and molecular characteristics			
Cellular senescence	High	Low	[26,33,81]

NS––No significant differences.

## Data Availability

No new data were created or analyzed in this study.

## Abbreviations

The following abbreviations are used in this manuscript:

aCGH	array Comparative Genomic Hybridization
ABCC2	ATP-binding cassette subfamily C member 2
AK	actinic keratosis
AMPK	5′ AMP-activated protein kinase
ANGPTL2	angiopoietin-349 like protein 2
APC	antigen presenting cells
ASC	adipose stromal/stem cells
AXL	tyrosine kinase receptors
BCC	basal cell carcinoma
BMP2	bone morphogenetic protein 2
BRAF	B-Raf proto-oncogene, serine/threonine kinase
CA14	Carbonic Anhydrase 14
CAF	cancer-associated fibroblasts
CDKN	cyclin-dependent kinase inhibitor
CpG	cytosine–guanine base
CNV	copy number variation
CRGs	circadian rhythm genes
CTLA-4	Cytotoxic T-lymphocyte associated protein 4
CXCR4	C-X-C Chemokine Receptor Type 4
DAMPs	damage-associated molecular patterns
DNAm	DNA methylation
ECM	extracellular matrix
ECOG	Eastern Cooperative Oncology Group
EGR3	Early Growth Response 3
EV	extracellular vesicles
EZH2	enhancer of zeste homolog 2
FBXW7	F-box and WD repeat domain containing 7
FMS	Familial Melanoma Syndrome
GRP78	78-kilodalton glucose-regulated protein
H2AX	H2A histone family member X
H3K4me1/ H3K9me3	Histone H3 methylation
HAPLN1	Hyaluronan And Proteoglycan Link Protein 1
HDAC	histone deacetylases
HRLs	hypoxia-related lncRNAs
hTERT	telomerase reverse transcriptase
ISI	immunosenescence index
IRE1	Inositol-requiring enzyme 1
KC	keratinocyte cancers
LDHB	Lactate Dehydrogenase B
LDH	lactate dehydrogenase
LEP	leptin
lncRNA	long noncoding RNA
Mfn	mitofusin
MMP	metalloproteinase
mTORC1	Mammalian Target of Rapamycin Complex 1
mtDNA	mitochondrial DNA
NF-κB	Nuclear factor kappa-light-chain-enhancer of activated B cells
NMSC	non-melanoma cancers
NRAS	Neuroblastoma RAS Viral Oncogene Homolog
OS	overall survival
p16INK4a	inhibitor of cyclin-dependent kinase 4
PD-1	Programmed cell death protein 1
PN	proteostasis network
P-P53	tumor antigen p53
PSEN2	presenilin 2
pTNM	pathological tumor-node-metastasis staging
RB1	RB Transcriptional Corepressor 1
RFS	recurrence-free survival
ROS	reactive oxygen species
RNA-seq	RNA sequencing
S1pr1	sphingosine-1-phosphate receptor 1
SASP	senescence-associated secretory phenotype
SCC	squamous cell carcinoma
SETD2	SET Domain-Containing Protein 2
SIRT1	Sirtuin 1
SM	skin melanomas
SMAD	Suppressor of Mothers Against Decapentaplegic
snoRNAs	Small nucleolar RNAs
SQ	squalene
TCGA	The Cancer Genome Atlas
TERT	telomerase reverse transcriptase
TGFβ	transforming growth factor
TKIs	tyrosine kinase inhibitors
TIL	tumor infiltrating lymphocytes
TME	tumor microenvironment
TMB	tumor mutational burden
TNFα	tumor necrosis factor alpha
UV	ultraviolet radiation
